# Apical Takotsubo Syndrome After Elective Stenting of the Left Anterior Descending Artery

**DOI:** 10.1016/j.jaccas.2026.108054

**Published:** 2026-04-28

**Authors:** Ksenia Vitalieva, Yuliya Shalaginova, Olga Stukalova, Igor Yavelov, Dmitry Pevzner

**Affiliations:** a Department of Acute Cardiology, E.I. Chazov National Medical Research Center of Cardiology, Moscow, Russia; b Department of Tomography, E.I. Chazov National Medical Research Center of Cardiology, Moscow, Russia; c Department of Fundamental and Clinical Problems of Thrombosis in Non-Communicable Diseases, National Medical Research Center for Therapy and Preventive Medicine, Moscow, Russia

**Keywords:** acute coronary syndrome, cardiomyopathy, coronary angiography, imaging, myocardial infarction, percutaneous coronary intervention

## Abstract

**Background:**

Takotsubo syndrome (TS) is a transient stress-induced left ventricular dysfunction that may clinically mimic acute myocardial infarction (MI). Its occurrence after percutaneous coronary intervention is rare, and it may be misinterpreted as a procedural complication, including type 4a MI.

**Case Summary:**

We describe a 77-year-old woman with stable coronary artery disease who developed apical TS shortly after elective stenting of the left anterior descending artery. Postprocedural chest pain and electrocardiographic changes raised suspicion of acute MI. However, coronary angiography demonstrated patent stent implantation without evidence of acute thrombosis. Cardiac magnetic resonance imaging confirmed apical ballooning with myocardial edema in the absence of necrosis, consistent with TS.

**Discussion:**

TS after percutaneous coronary intervention is an uncommon but important diagnostic consideration. Differentiation from periprocedural MI requires integration of clinical findings, coronary imaging, and cardiac magnetic resonance imaging. Recognition of this condition is essential to avoid unnecessary escalation of antithrombotic therapy and to guide appropriate management.

## Visual Summary

**Figure:**
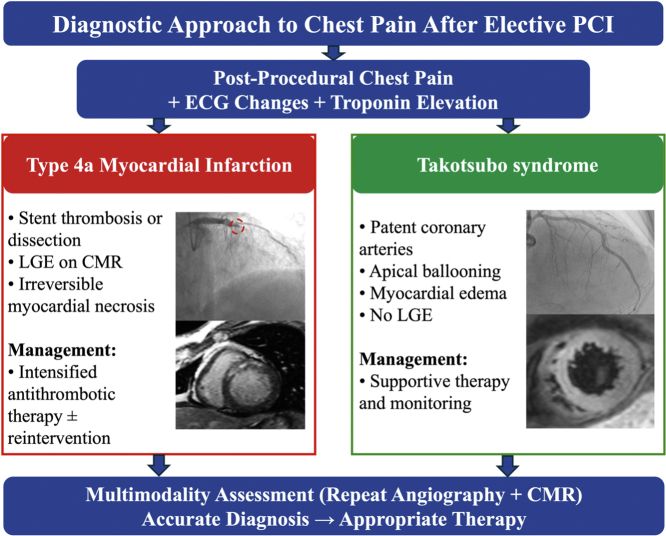

**Visual Summary:**
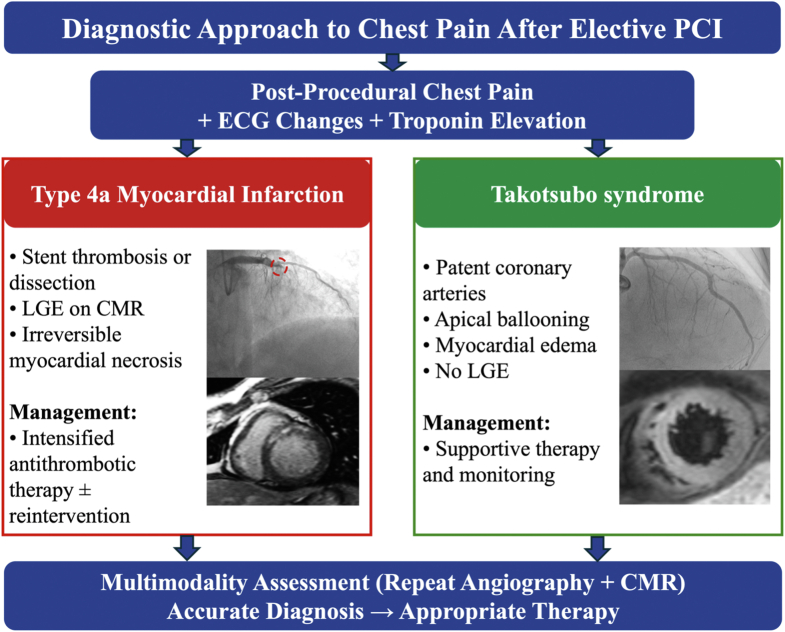
Diagnostic Approach to Chest Pain After Elective Percutaneous Coronary Intervention This algorithm illustrates the differential diagnostic pathway in a patient presenting with chest pain and biomarker elevation after elective left anterior descending artery stenting. Key distinguishing features between type 4a myocardial infarction and Takotsubo syndrome are highlighted, emphasizing the pivotal role of repeat coronary angiography and cardiac magnetic resonance imaging in establishing the correct diagnosis and guiding management. CMR = cardiac magnetic resonance imaging; ECG = electrocardiogram; LGE = late gadolinium enhancement; PCI = percutaneous coronary intervention.

Takotsubo syndrome (TS) is a reversible left ventricular (LV) myocardium systolic dysfunction, with clinical manifestations that in most cases overlap with those of an acute myocardial infarction (MI).^1^ First described in Japan in the 1990s, it is characterized by transient regional wall motion abnormalities in the absence of obstructive coronary disease explaining the extent of myocardial dysfunction. The typical ventricular configuration resembles an octopus trap (“tako-tsubo”).

The prevalence of TS is estimated at 1% to 3% of all acute coronary syndrome cases and 0.5% to 0.9% of ST-segment elevation MI presentations.^1^ TS is more common in women, estimated at 2% to 3% of female patients with ST-segment elevation acute coronary syndrome, with up to 90% of TS cases occurring in postmenopausal women.^2^

Clinical manifestations include chest pain, dyspnea, and/or syncope. Asymptomatic TS cases have also been described. In severe cases, complications such as heart failure, pulmonary edema, stroke, and cardiogenic shock may develop.

Areas of impaired contractility of the left ventricle (LV; or both ventricles) in TS extend beyond the regions known to be supplied by specific coronary arteries, apparently matching segments of sympathetic innervation. The typical (apical) phenotype is the most common (80%), while midventricular (14%-17%), basal (1%-2.2%), and focal (1%) variants are less frequent. The right ventricle is affected in about one-third of TS cases.^3^

The most widely accepted mechanism involves excessive catecholamine release leading to myocardial stunning, microvascular dysfunction, and direct myocardial toxicity.^4^ The higher density of beta-adrenoreceptors in the apical segments may explain the typical pattern of apical hypokinesis.

## History of Presentation

**Figure 1:**
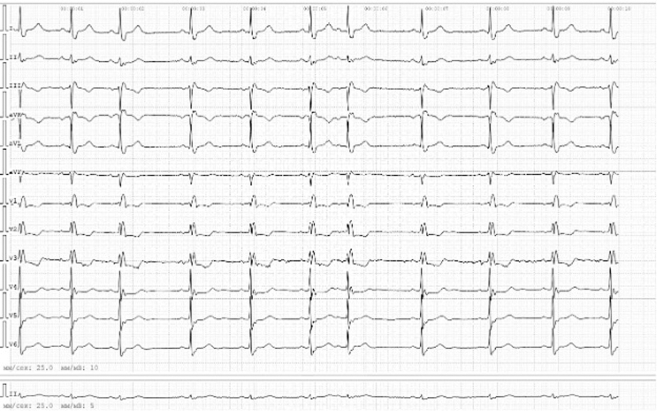
Electrocardiogram on Admission Electrocardiogram indicated sinus arrhythmia with heart rate of 63 to 69 beats/min, left anterior fascicular block, and right bundle branch block.

A 77-year-old woman presented on April 11, 2024, to the E.I. Chazov National Medical Research Center of Cardiology for elective hospitalization, with clinical symptoms of angina at exertion consistent with NYHA functional class III. On admission, her blood pressure was 125/70 mm Hg and her heart rate was 74 beats/min. Physical examination revealed no signs of heart failure. Baseline electrocardiography showed sinus rhythm with left anterior fascicular block and a JT interval of 320 ms (Figure 1).

## Past Medical History

The patient had a history of hypertension treated with perindopril 5 mg and amlodipine 5 mg daily, with well-controlled blood pressure. There was no prior history of MI or stroke. She was taking aspirin 75 mg daily. The patient was under observation by a psychiatrist for emotional lability personality disorder, without medications.

## Investigations

Transthoracic echocardiography on April 3, 2024, indicated the cardiac chambers were not enlarged, walls were not thickened, and there were no regional wall motion abnormalities; the ejection fraction (EF) was 60%. Moderate aortic stenosis and grade 1 aortic regurgitation were detected. Chest radiography was unremarkable.

Stress echocardiography (50 W) demonstrated the appearance of a hypokinesis area along the anterior wall (mid and apical segments) extending to the apical segment of the anteroseptal LV wall. At maximum exertion, the patient reported a sensation of tightness in her chest.

**Figure 2:**
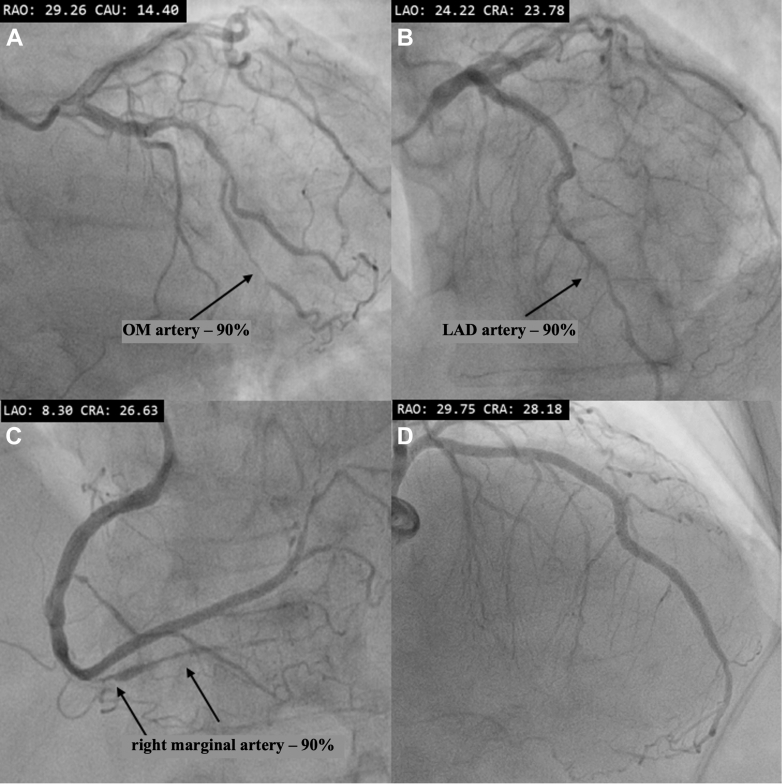
Coronary Angiography Coronary angiography demonstrated (А) a 90% lesion in the obtuse marginal artery, diameter ∼2 mm (arrow), (В) a 90% lesion in the midsegment of the LAD (arrow), and (С) several lesions up to 90% in the right marginal artery, diameter ∼2 mm (arrows). (D) Outcome of balloon angioplasty with stenting of the LAD midsegment; in total, 2 segments were implanted. LAD = left anterior descending artery; OM = obtuse marginal.

Considering the available findings, diagnostic coronary angiography (CAG) was performed and demonstrated a predominantly right-sided coronary blood supply pattern, with 90% lesion in the midsegment of the left anterior descending artery (LAD), 50% lesion in the mid-third of the circumflex artery, 90% lesion in the obtuse marginal artery (diameter: ∼2 mm), and jagged lining of the right coronary artery with some lesions up to 90% in the right marginal artery (diameter: ∼2 mm) (Figures 2A to 2C).

Given the hemodynamically significant LAD lesion, percutaneous coronary intervention (PCI) was performed with implantation of 2 Synergy stents (2.5 × 28 mm and 3.0 × 28 mm; Boston Scientific), resulting in angiographical success (TIMI flow grade III) (Figure 2D). A loading dose of clopidogrel 600 mg was administered before PCI.

**Figure 3:**
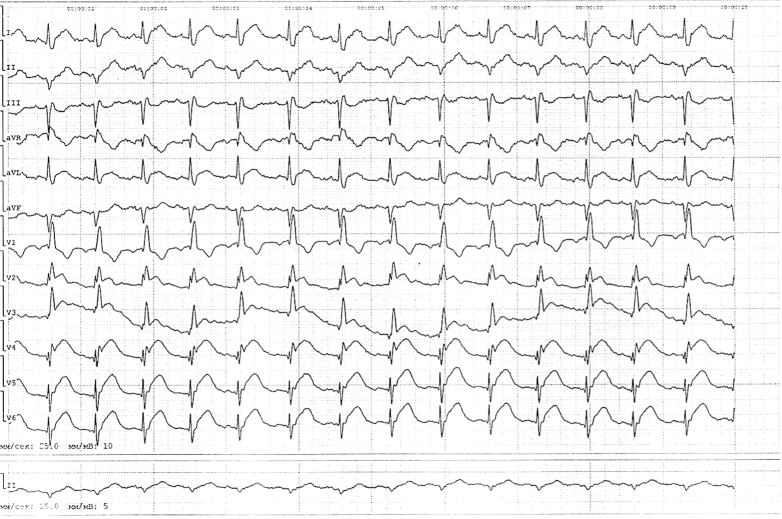
Electrocardiogram During Pain Electrocardiogram showed sinus rhythm at 100 beats/min, ST-segment elevation up to 2 mm in leads I, II, aVL, and V_2_–V_6_, and possible ST-segment depression up to 1 mm in lead aVR; interpretation is limited by the underlying bifascicular block.

During balloon inflation in the LAD, the patient developed intensive pressure-like chest pain lasting up to 3 minutes with ST-segment depressions up to 2 mm in leads II-III. The patient was transferred in stable condition to the intensive care ward for follow-up observation. Thirty minutes later, she developed anginal pain accompanied by ST-segment elevation up to 2 mm in leads I, II, aVL, and V_2_-V_6_; the JT interval was 320 ms (Figure 3).

**Figure 4:**
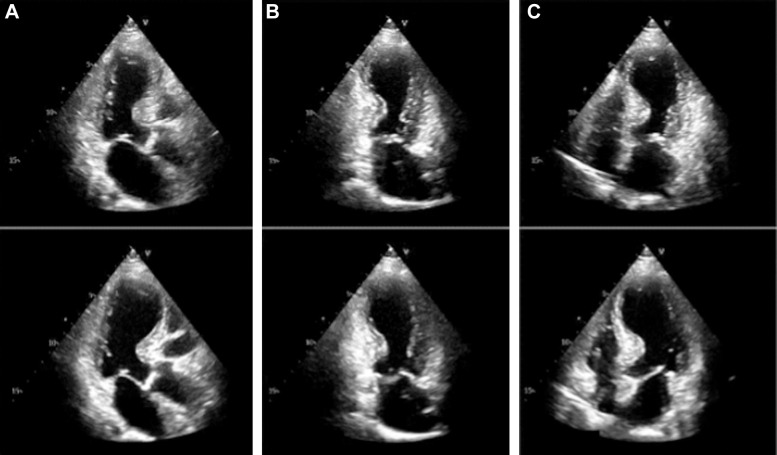
Bedside Echocardiography (Cardiac Point-of-Care Ultrasound) During the Acute Phase of Takotsubo Syndrome The upper row shows left ventricle images during systole, the lower row shows images during diastole. (А) Apical 3-chamber view, (В) apical 2-chamber view, and (С) apical 4-chamber view. Ejection fraction was reduced to 47%, and hypokinesis of the left ventricle apex, apical segments of the anterior, inferior walls, and the interventricular septum was observed.

Echocardiography demonstrated new LV systolic dysfunction (EF: 48%) with hypokinesis of the apex and apical segments of the anterior and inferior walls and the interventricular septum (Figure 4).

Repeat CAG showed recently implanted stents without thrombosis, dissection, perforation, or distal embolization; TIMI flow grade III and normal myocardial blush (grade 3) were confirmed.

The chest pain resolved spontaneously within 15 minutes. High-sensitivity troponin peaked at 4,535 pg/mL (reference <15.6 pg/mL, not measured on admission), N-terminal pro–B-type natriuretic peptide (NT-proBNP) increased to 2,968 pg/mL (reference: <125.0 pg/mL, on admission: 94 pg/mL).

Taking into account the clinical findings, such as the development of a typical anginal attack preceded by a stress trigger (emotional and physical stress), evidence of LV myocardium impairment and dysfunction (ECG findings, apical area of impaired local contractility on echocardiography consistent with a typical TS phenotype, not matching coronary artery supply areas, laboratory elevation of cardiac-specific markers), lack of thrombosis or dissection of coronary arteries or evidence of distal coronary embolization on follow-up CAG, the diagnosis of TS was established.

## Differential Diagnosis

The differential diagnosis included: type 4 MI (4a and 4b: periprocedural injury or stent thrombosis) and TS.

## Management

Treatment included initiation of bisoprolol 1.25 mg once daily and losartan 50 mg once daily (dose titration limited by borderline blood pressure). Dual antiplatelet treatment (aspirin 100 mg and clopidogrel 75 mg daily) and atorvastatin 40 mg daily were continued.

## Outcome and Follow-Up

On day 3, ECG demonstrated persistent ST-segment elevation in leads V_2_-V_6_ with evolving T-wave inversion; the JT interval was within 320 ms.

**Figure 5:**
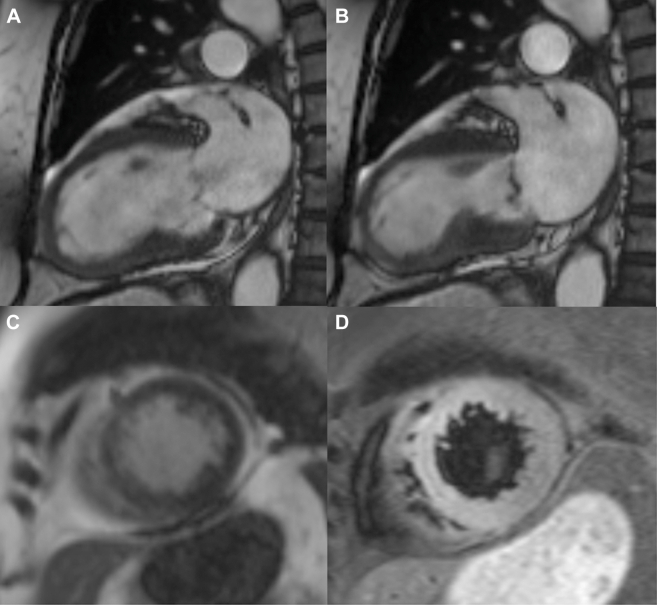
Contrast-Enhanced CMR on Day 7 After Takotsubo Syndrome Onset (А) Cine image, 2-chamber slice, diastolic phase. (В) Cine image, 2-chamber slice, systolic phase. Hypokinesis of the apical LV segments is observed. (С) CMR with delayed contrast enhancement, LV short axis at the level of apical segments shows no gadolinium uptake in the myocardium. (D) Т2-weighed image. LV short axis at the level of apical segments. Edema of the interventricular septum extending to the LV anterior wall. CMR = cardiac magnetic resonance imaging; LV = left ventricle.

Cardiac magnetic resonance imaging (CMR) performed on day 7 showed an LVEF of 50% and persistent apical hypokinesis (apex and apical segments of the anterior and inferior walls and the interventricular septum), which was inconsistent with the areas supplied by the LAD or other coronary arteries. Т2-weighed imaging demonstrated edema of the interventricular septum extending to the anterior LV wall in the apical and midsegments; no contrast accumulation was found in the ventricles. Diagnosis was confirmed (Figure 5).

During the observation, the treatment administered resulted in a subjective improvement of the patient's overall well-being. Chest pain did not recur, and there was no dyspnea on physical exertion, compatible with the in-hospital stay. Serial troponin levels showed a declining trend (3,273 pg/mL on day 4, 710 pg/mL on day 7, and 67 pg/mL on day 10). By day 10, the ST segment returned to baseline in leads V_2_-V_5_, negative Т waves were formed in all chest leads, and the JT interval was 320 ms. Echocardiography demonstrated partial recovery of LV function (EF: 52%), with residual apical hypokinesis. The patient was discharged on day 11 in stable condition.

**Figure 6:**
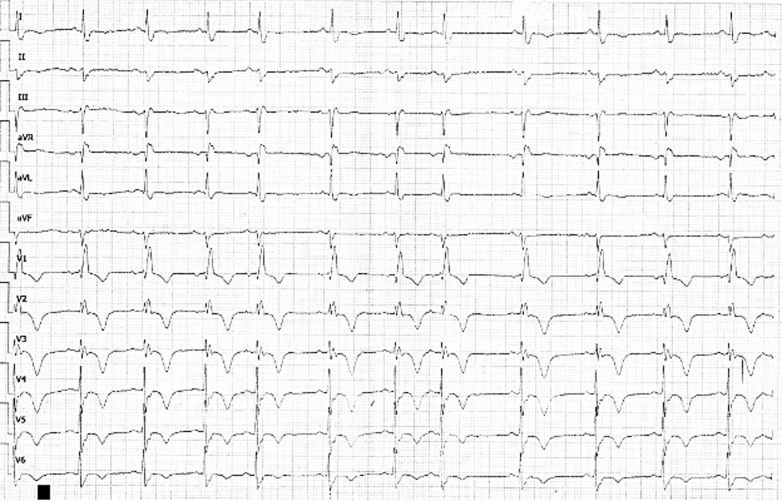
Electrocardiogram at 6 Weeks After Discharge Sinus arrhythmia, heart rate ∼71 beats/min, bifascicular block, ST-segment in leads V_2_-V_6_ is at baseline, negative T waves in chest leads, JT interval within 320 ms.

At the 6-week follow-up, high-sensitivity troponin normalized (15.2 pg/mL), NT-proBNP decreased to 781.4 pg/mL, and echocardiography showed full recovery of LV systolic function (EF: 60%) without regional wall motion abnormalities. ECG demonstrated persistent T-wave inversion (Figure 6).

## Discussion

PCI with stenting, like other invasive procedures, increases sympathetic activity and release of catecholamines, acting as an emotional and physical trigger of TS development.^5^^,^^6^ The onset of an anginal attack early after PCI requires differential diagnosis between TS and types 4а or 4b MI.

The patient's characteristics were most typical for TS: an elderly postmenopausal woman with emotional lability personality disorder.

ECG findings in TS are nonspecific. According to the International Takotsubo registry (InterTAK), the most common ECG findings are ST-segment elevation (44%) and depression (8%), Т-wave inversion (41%), lack of reciprocal findings, and left bundle branch block (5%). Some reports suggest highly specific ECG findings to be used for TS diagnosis, such as lack of abnormal Q waves, ST-segment depression in aVR, and lack of nonsignificant (within 1 mm) ST-segment elevation in V_1_; such ECG findings demonstrate a sensitivity of 91%, specificity of 95%, and diagnostic precision of 95% for TS diagnosis.^7^

In the present case, ST-segment elevation was observed in leads I, II, aVL, and V_2_- V_6_ without ST-segment elevation in lead V_1_ and reciprocal changes, findings consistent with previously described TS patterns.

QT prolongation is another relatively common ECG manifestation of TS, reported in 26% to 50% of cases.^8^ Although prolonged repolarization may predispose to ventricular arrhythmias, its prognostic significance remains uncertain. Because the patient had bifascicular block, the JT interval was used to assess repolarization and remained within normal limits during hospitalization.

In TS, the area of myocardial damage does not match regions supplied by any specific coronary artery. In this case, echocardiography demonstrated hypokinesis involving the LV apex and apical segments of all walls, a pattern typical for the apical phenotype of TS and not confined to the distribution of the LAD.

Myocardial damage in TS is associated with elevation of troponin and NT-proBNP. In TS, the moderate magnitude of troponin elevation is disproportional to the extensive area of damaged LV myocardium area, and typically, the NT-proBNP/troponin ratio in TS is higher than in acute MI.

Peri- and postinterventional complications resulting in acute injury and type 4 MI are explained by multiple factors: stent thrombosis, occlusion of lateral branches, distal embolism with no/slow-reflow phenomenon, coronary artery dissection, vasospasm. According to the InterTAK diagnostic algorithm, as proposed in the consensus statement of 2018,^9^ coronary imaging is recommended if ST-segment elevations are identified on ECG, to support differential diagnosis between TS and MI.

CMR plays a crucial role in confirming the diagnosis. Typical findings include edema on Т2-weighed imaging without late gadolinium enhancement, reflecting reversible myocardial injury. In the present case, CMR demonstrated myocardial edema involving apical and midventricular segments without gadolinium accumulation, supporting the diagnosis of TS.

Although TS was previously considered a benign condition, contemporary studies have shown that serious complications may occur during the acute phase. Cardiogenic shock develops in approximately 9% to 11% of patients, and LV outflow tract obstruction has been reported in up to one-fourth of cases. The severity and prognosis of TS have been found to depend on the nature of the trigger: emotional, physical, or undetermined. Physical triggers, surgical interventions in particular, and traumatic injuries are independent predictors of poor outcomes, such as short- and long-term mortality, the rate and severity of heart failure, and ventricular arrhythmias. In this patient, the PCI procedure itself likely acted as a physical trigger, while procedural anxiety may also have contributed.

There is currently no specific pharmacological therapy proven to improve outcomes in TS, and treatment is largely supportive. Beta-blockers and angiotensin-converting enzyme inhibitors or angiotensin receptor blockers are commonly used during the acute phase, although evidence for long-term benefit remains limited and is largely derived from observational studies.^10^^,^^11^

In the present case, the patient experienced an uncomplicated clinical course with gradual recovery of LV systolic function, consistent with the reversible nature of TS, 6 weeks after discharge, and complete recovery of LV contractility was documented.

## Conclusions

In patients presenting with chest pain and ST-segment elevation early after PCI, urgent repeat coronary angiography is essential to exclude procedural complications. CMR plays a key role in the differential diagnosis by demonstrating myocardial edema in the absence of late gadolinium enhancement.

In the present case, distal embolization of the LAD with spontaneous thrombus lysis cannot be completely excluded. However, the extent of myocardial edema exceeded the vascular territory supplied by the distal LAD, and repeat coronary angiography revealed no evidence of embolization. These findings make Takotsubo syndrome the most likely diagnosis.

Evidence regarding pharmacological therapy remains limited. Nevertheless, current international recommendations support the use of angiotensin-converting enzyme inhibitors or angiotensin receptor blockers, particularly in patients with impaired left ventricular systolic function.

## Funding Support and Author Disclosures

The authors have reported that they have no relationships relevant to the contents of this paper to disclose.

**Take-Home Messages**

• Takotsubo syndrome should be considered in patients with chest pain after PCI when angiography excludes procedural complications.
• Cardiac magnetic resonance imaging is essential for differentiating Takotsubo syndrome from type 4a myocardial infarction and excluding stent thrombosis.
